# Schizophrenia and eating disorders: Epidemiological and clinical characteristics

**DOI:** 10.1192/j.eurpsy.2024.1154

**Published:** 2024-08-27

**Authors:** E. Herelli, M. Lagha, F. Askri, I. Ben Romdhane, H. Wided, R. Labbane

**Affiliations:** PSYCHIATRY C, RAZI HOSPITAL, MANOUBA, Tunisia

## Abstract

**Introduction:**

Schizophrenia is a common mental illness with a wide range of symptoms.

Given the high metabolic comorbidity observed in schizophrenia and the metabolic side-effects induced by the antipsychotics used in practice, the detection of eating disorders is crucial.

These disorders may occur at the same time as psychotic symptoms or independently of them.

**Objectives:**

we aim to provide an overview of eating disorders in schizophrenia.

**Methods:**

We conducted a systematic search using the 2 bibliographic databases PubMed and Google scholar including the following keywords: “Schizophrenia”, “Eating disorders”, “Reward mechanisms”.

**Results:**

Eating disorders are a frequent comorbidity in schizophrenia.

Authors have reported that some patients with schizophrenia have an increased appetite and craving for fatty foods, increased caloric intake and frequency of consumption, which may be associated with increased disinhibition.

According to the literature, binge eating and night eating are frequently observed in patients with schizophrenia, with a prevalence of around 10%.

Studies have shown that people suffering from psychosis and treated with antipsychotics have high rates of binge eating, ranging from 4.4% to 16% for binge eating and from 8.9% to 45% for binge eating symptoms (without reaching the diagnostic threshold for binge eating).

Rates ranging from 16.1% to 64% for cravings were reported.

Anorexia nervosa appears to affect between 1% and 4% of schizophrenic patients.

**Conclusions:**

Despite their frequent association with schizophrenia, eating disorders remain little studied. However, it is important to detect these disorders and elucidate the underlying psychopathological and neurobiological mechanisms in order to better manage metabolic comorbidity and improve patients’ quality of life.

**Disclosure of Interest:**

None Declared

